# Ecological momentary assessment and applied relaxation: Results of a randomized indicated preventive trial in individuals at increased risk for mental disorders

**DOI:** 10.1371/journal.pone.0286750

**Published:** 2023-06-08

**Authors:** Eva Asselmann, Monique Zenker, Frank Rückert, Hanna Kische, Lars Pieper, Katja Beesdo-Baum

**Affiliations:** 1 Department of Psychology, Institute for Mental Health and Behavioral Medicine, HMU Health and Medical University, Potsdam, Germany; 2 Faculty of Psychology, Institute of Clinical Psychology and Psychotherapy, Behavioral Epidemiology, Technische Universität Dresden, Dresden, Germany; Osaka Metropolitan University: Osaka Koritsu Daigaku, JAPAN

## Abstract

Applied Relaxation (AR) is an established behavioral mental health intervention, but its efficacy in real life contexts remains unclear. Using randomized controlled trial data, we examined whether AR can effectively reduce mental health problems in daily life. A sample of 277 adults with increased psychopathological symptoms but without 12-month DSM-5 mental disorders at study entry was randomly assigned to an intervention group receiving AR training (n = 139) and an assessment-only control group (n = 138). Ecological momentary assessments were used to assess psychological outcomes in daily life over a period of seven days at baseline, post, and 12-month follow-up, respectively. Multilevel analyses indicated that all psychopathological symptoms decreased more in the intervention group than in the control group from baseline to post (range *β =* -0.31 for DASS-depression to *β =* -0.06 for PROMIS-anger). However, from post to follow-up, psychopathological symptoms decreased more in the control group than in the intervention group, so that only the intervention effects for PROMIS-depression (*β =* -0.10) and PROMIS-anger (*β =* -0.09) remained until follow-up. Moreover, positive affect (*β =* 0.19), internal control beliefs (*β =* 0.15), favorable coping (*β =* 0.60), and unfavorable coping (*β =* -0.41) improved more in the intervention group than in the control group, and these effects were mostly maintained in the long term. Some effects were stronger among women, older individuals, and individuals with higher initial symptoms. These findings suggest that AR can effectively reduce mental health problems in daily life.

**Trial registration.** The trial has been registered at ClinicalTrials.gov (NCT03311529).

## Introduction

Mental disorders are common and rank among the top causes and risks for years lived with disability around the world. They relate not only to individual impairments but also to tremendous societal costs [1–7]. An important task in clinical psychology is therefore to develop targeted interventions that can not only treat mental disorders but also prevent them as soon as the very first symptoms occur.

Cognitive-behavioral models suggest that psychophysiological tension plays an important role in a cascade of symptoms that reinforce each other and can result in full-threshold mental disorders over time [8,9]. Relaxation interventions aim to break this cycle. They lower psychophysiological tension, which in turn promotes improvements in other psychophysiological (e.g., lower heart rate), emotional (e.g., lower depressive, anxiety, and stress symptoms), cognitive (e.g., higher perceived control and higher self-efficacy), and behavioral (e.g., more favorable and less unfavorable coping) outcomes.

A well-known relaxation technique is Applied Relaxation (AR). Originally developed to treat anxiety disorders, it is conceptualized as a behavioral coping technique that enables individuals to rapidly relax within 20 to 30 seconds as soon as the first signs of tension or stress appear [10]. AR can be trained individually or in small groups. Individuals first learn to perform Progressive Muscle Relaxation. Then they gradually practice relaxing in shorter intervals, recognizing the first signs of tension at an early stage, and relaxing not only in stress-free but also in imaginary and real stressful situations. In contrast to other relaxation techniques (e.g., Progressive Muscle Relaxation), AR specifically promotes the transfer from the training setting to challenging episodes in daily life. The technique is thus predestined to reduce psychopathologically symptoms in daily life and prevent a symptom escalation over time [11].

As single treatment or part of a multimodal program, AR has been shown to effectively reduce depressive, anxiety, stress, or somatic symptoms in the context of various mental disorders and physical diseases. For instance, AR reduced anxiety symptoms in patients with generalized anxiety disorder [12–16], panic disorder [11,17,18], agoraphobia [18–20], social phobia [21–23], and specific phobias [24,25]. AR has also been shown to reduce the intensity and duration of pain in patients with tinnitus [26,27], chronic or recurrent headache [28–30], migraine [31,32], neck/back [33,34], or longstanding/chronic pain [35–38]. Furthermore, AR had favorable effects among non-patient groups such as pregnant women [39], students [40–42], and athletes [43]. However, to our knowledge, no previous study has investigated whether AR leads to symptom improvements in time to experience in real-life contexts. In this regard, studies with embedded ecological momentary assessments (EMA) promise to be particularly useful because EMA minimize retrospective recall biases and increase the accuracy, ecological validity, and generalizability of self-report data [44].

A randomized controlled trial recently investigated whether AR lowers existing psychopathological symptoms and thus prospectively prevents the onset of subthreshold and full-threshold mental disorders in the following months [45,46]. Participants with elevated depressive, anxiety, or stress symptoms but without full-threshold mental disorders at study entry were randomly assigned to an assessment-only control group or to an intervention group that received group-based AR training. At baseline, post, and 12-month follow-up, psychopathological symptoms were assessed with conventional questionnaires and with smartphone-based EMA in daily life.

The conventional questionnaires at the respective main assessment revealed that the primary (21-item short-form of the Depression Anxiety Stress Scale, DASS-21) and secondary (depressive, anxiety, anger, and somatic symptoms as well as sleep disturbances assessed with the DSM-5 Level 2 Cross-Cutting Symptom Measures) outcomes of the intervention efficacy improved more in the intervention group than in the control group from baseline to post. However, these group effects did not persist until follow-up. That is, AR led to accelerated improvements in various psychopathological symptoms. In addition, a reduction of incident (sub)threshold mental disorders as primary outcome of the prevention efficacy was observed.

### Aims

The current analysis addresses secondary research questions from this RCT [45]. The aims are to examine whether AR significantly reduces psychopathological symptoms from baseline to post and from baseline to follow-up, captured via EMA in time to experience five times daily for one week at each wave. Moreover, we aim to investigate whether AR leads to improvements in health-related emotional (i.e., more positive affect), cognitive (i.e., higher perceived control and higher self-efficacy), and behavioral (i.e., more favorable and less unfavorable coping) traits from baseline to post and from baseline to follow-up.

Finally, we aim to examine whether the intervention efficacy varies by sex, age, and initial symptom severity at baseline. Investigating such moderator effects is crucial for specifying high-risk groups that might particularly benefit from AR training. According to theories of motivation [47] and health behavior [48], individuals are more likely to change their behavior if they perceive that change to be important. Consistent with this idea, it is plausible to assume that participants with more severe symptoms are more motivated to learn AR, more compliant during the intervention, and more likely to use AR in daily life [26]. In addition, there is evidence that relaxation training tends to be more effective in younger (vs. older) individuals and in women (vs. men) [49]. Thus, we hypothesize that the intervention efficacy will be stronger in younger individuals, in women, and in participants with higher symptom severity at baseline.

The current study does not overlap with previous work [45] because it is based on different data (from the EMA) and focuses (in part) on different research questions and hypotheses (e.g., changes in health-related emotional, cognitive, and behavioral traits as well as moderator effects).

## Materials and methods

### Design

A randomized controlled parallel-group superiority trial with an intervention group and an assessment-only control group was conducted from 9/2016 to 11/2019 at the Institute of Clinical Psychology and Psychotherapy, Technische Universität Dresden, Germany [45]. Participants in the intervention group received group-based AR training, whereas participants in the control group did not receive any intervention. Participants were assigned to the intervention or control group via balanced randomization [ratio 1:1] based on computer-generated permutated blocks by the principal investigators (KBB and/or EA).

The trial included a baseline, post, and 12-month follow-up assessment. The post assessment was conducted directly after the intervention in the intervention group and after a comparable time frame (i.e., approximately 3 months after baseline) in the control group. The follow-up assessment was conducted approximately 12 months after the post assessment. Conditions were not blinded.

The authors assert that all procedures contributing to this work comply with the ethical standards of the relevant national and institutional committees for experiments on humans and the Helsinki Declaration of 1975 as amended in 2013. The study was approved by the ethics committee of the Technische Universität Dresden (EK508112015). All participants gave written informed consent after being completely informed about the study. They received an expense allowance of 8.50 euros per hour for their participation in the post and follow-up assessment, respectively.

### Participants

Participants were recruited via flyers, advertisement, and media articles from the general population and cooperating institutions in the Dresden area. Individuals interested in the study were screened for initial inclusion criteria (see below) via a secured webpage hosted by the Technische Universität Dresden. Individuals who met these criteria were invited to a face-to-face entry exam, during which a standardized diagnostic interview modified for DSM-5 (DIA-X-5) [50] was conducted.

Inclusion criteria were at least mild depressive, anxiety, or stress symptoms (DASS-21 depression score ≥ 5, anxiety score ≥ 4, or tension/stress score ≥ 8) [51] and an age between 18 and 55 years. The main exclusion criterion was a 12-month diagnosis of a mental disorder, including somatic symptom and related disorders (i.e., somatic symptom disorder and illness anxiety disorder), anxiety disorders (i.e., panic disorder, generalized anxiety disorder, social anxiety disorder, agoraphobia, and separation anxiety disorder), depressive disorders (i.e., major depression and persistent depressive disorder/dysthymia), bipolar disorders (i.e., bipolar I and bipolar II disorder), posttraumatic stress disorder, and substance use disorders (i.e., alcohol use disorder, medication use disorders, and illicit substance use disorders). Additional exclusion criteria were psychotic symptoms and acute suicidality. It was planned that individuals experiencing psychosis or acute suicidality during the study would be withdrawn from the study and referred to treatment. However, no such incidents occurred. Participants were required to not receive any pharmacological or psychological interventions at study entry but were free to seek such (additional) interventions during the study. Actual treatment seeking was assessed at follow-up.

### Intervention

The AR intervention was manualized following the procedures of Öst [10,52] and conducted in group format with approximately 10 participants per group. Each course consisted of 10 instructor-guided sessions (each lasting about one hour) with the following content: (1) Psychoeducation (i.e., information on the rationale, aims, and procedures of AR) and Progressive Muscle Relaxation, (2) release-only relaxation I (i.e., direct muscle relaxation without prior tension), (3) release-only relaxation II, (4) cue-controlled relaxation (i.e., relaxation with a personal cue-word), (5) differential relaxation I (i.e., relaxation with eyes open and with movement of the eyes, head, arms, or legs, and while sitting), (6) differential relaxation II (i.e., relaxation while standing and walking), (7) rapid relaxation (i.e., shortened cue-controlled relaxation [20–30 seconds] in daily life), (8) AR—imaginal practice (i.e., rapid relaxation upon noticing first signs of tension triggered by an imaginary scenario), (9) AR—real life practice (i.e., transfer to real-life situations typically associated with psychophysical, emotional, cognitive, or behavioral symptoms of tension or stress), (10) AR in real life, relapse prevention, and closing.

The course sessions were accompanied by weekly homework (i.e., daily practice of the relaxation exercise with corresponding notes in a relaxation diary). In addition, participations were instructed to document daily stressful episodes and associated psychophysical, cognitive, behavioral, and/or emotional symptoms in a tension/stress diary (to practice early recognition of initial signs of tension). Homework assignments were prepared and discussed in each course session. All course instructors (*N* = 4) had a psychology study background and were trained in AR by the principal investigators (KBB and/or EA) and supervised throughout the study. The AR course manual and materials are available upon request from KBB or EA.

### Outcome measures

At baseline, post, and follow-up, psychopathological symptoms and other emotional (i.e., positive affect), cognitive (i.e., perceived control and self-efficacy), and behavioral (i.e., favorable and unfavorable coping) outcomes were assessed via EMA. That is, participants were given a smartphone and instructed to answer a battery of items in time to experience five times a day for one week. Individual assessments were scheduled based on a time sampling scheme with variable assessment intervals. To keep the EMA short and feasible, individual scales were slightly abbreviated. Some items were slightly reworded so that they referred to the time interval after the last assessment (e.g., “in the last two weeks” was changed to “since the last assessment”). Items were answered with a slider-response ranging from 0 to 100. Items are available upon request from KBB or EA.

In the evening, depressive, anxiety, and stress symptoms on the respective day were assessed with the DASS-21 [51], which was also used to assess the primary outcome at the respective main assessment. The response scale ranged from 0 = “not at all” to 100 = “all the time”.

Depressive, anxiety, anger, and somatic symptoms as well as sleep disturbances since the last assessment were assessed with the DSM-5 Level 2 Cross-Cutting Symptom Measures [53,54]). More specifically, the Patient-Reported Outcomes Measurement Information System (PROMIS) short forms for Emotional Distress (i.e., depression, anxiety, anger) [55] and Sleep Disturbance [56] as well as an adapted version of the Patient Health Questionnaire 15 Somatic Symptom Severity Scale (PHQ-15) [57] were used. The DSM-5 Level 2 Cross-Cutting Symptom Measures were applied five times daily, except for sleep disturbances, which were assessed only in the morning. The response scale ranged from 0 = “not at all” to 100 = “all the time” for PROMIS-depression, PROMIS-anxiety, and PROMIS-anger, from 0 = “not at all” to 100 = “very much” for PHQ-15-somatic symptoms, and from 0 = “very bad” to 100 = “very good” for PROMIS-sleep.

At each wave, positive affect since the last assessment was assessed with the respective subscale of the Positive and Negative Affect Schedule (PANAS) [58], labeled from 0 = “not at all” to 100 = “extremely”. Negative affect was not assessed in the EMA because respective items overlapped with the symptom measures and the EMA item battery was intended to be as short as possible.

In the evening, internal and external control beliefs on the respective day were assessed with the 4-item Internal External Control Scale (IE-4) [59] and self-efficacy on the respective day was assessed with the General Self-Efficacy Short Scale (ASKU) [60], labeled from 0 = “not at all” to 100 = “completely”.

In the evening, participants were also asked whether anyone or anything had bothered, upset, or disturbed them on that particular day. If participants affirmed this question, favorable (i.e., relaxation and positive self-instruction) and unfavorable (i.e., flight and avoidance) coping strategies in that situation(s) were measured with individual subscales of the Stress Coping Questionnaire [61], labeled from 0 = “not at all” to 100 = “entirely”.

### Primary and secondary outcome measures

In this trial, changes in depressive, anxiety, and stress symptoms (assessed with the DASS-21 at the respective main assessment) in the intervention vs. control group from baseline to post were defined as the primary outcome of the intervention efficacy. Rates of incident mental disorders in the intervention vs. control group from entry exam to follow-up were defined as the primary outcome of the prevention efficacy. Findings for these outcome measures have been previously presented [45].

Secondary outcomes of the intervention efficacy included changes in other symptom measures (e.g., those assessed with the DSM-5 Level 2 Cross-Cutting Symptom Measures at the respective main assessment and symptom measures assessed via EMA) and clinical changes (e.g., number of symptoms and diagnoses, impairment, and disability) in the intervention vs. control group from baseline to post. Secondary outcomes of the prevention efficacy included changes in depressive, anxiety, stress, and other symptoms as well as clinical changes in the intervention vs. control group from post to 12-month follow-up. No important changes to the methods or trial outcomes were made after trial commencement.

### Sample size calculation

As previously presented [45], we assumed a medium effect size of 0.4 from baseline to post based on prior evidence from selective and indicated preventive interventions targeting symptoms of depression, anxiety, or stress [62–64]. Specifying a correlation of 0.5 between baseline and post, an alpha level of .05, and a statistical power of 0.8 yielded 74 participants required per group. Specifying a dropout rate of 15% from baseline to post and 15% from post to follow-up yielded 103 participants required per group at baseline.

### Statistical analyses

Analyses were performed using Stata 15 [65]. Multilevel mixed-effects linear regression models with maximum likelihood (ML) estimations (“mixed” command in Stata) and measurement occasions (Level 1) nested within persons (Level 2) were used to examine the research questions. To test whether outcome changes (a) from baseline to post, (b) from post to follow-up, and (c) from baseline to follow-up differed by group, we simultaneously regressed the respective outcome on a timing variable, a group variable, and an interaction term of the timing and group variables. Only observations at the respective two time points were considered. For example, the analyses on outcome changes from baseline to post excluded observations at follow-up. The timing variable was coded as 0 at the first time point and 1 at the second time point (e.g., 0 at baseline and 1 at post in the analyses on outcome changes from baseline to post). The group variable was coded as 0 in the control group and 1 in the intervention group.

To test whether intervention effects varied by (a) sex, (b) age, and (c) levels of the respective outcome at baseline, a three-way interaction term was included from the timing variable, the group variable, and (a) sex, (b) age, or (c) levels of the considered outcome at baseline, respectively. The variable “sex” was coded as 0 in men and 1 in women.

Because some symptoms were left-skewed and their residuals were not normally distributed, all outcomes were log-transformed (log(x+1)). To enable comparisons between different outcome measures and groups, all log-transformed outcomes were further standardized across all waves (*M* = 0, *SD* = 1) based on the pooled standard deviation of the intervention and control group at baseline. The age variable (in years) was divided by 10 to ensure that the effects did not become too small to be presented rounded. All analyses were adjusted for sex and age. The alpha level was set at .05. The Benjamini-Hochberg procedure was used to reduce the false discovery rate and correct for multiple testing [66].

## Results

A total of 277 individuals (intervention group: *N* = 139; control group: *N* = 138) participated at baseline, 229 individuals (intervention group: *N* = 110; control group: *N* = 119) participated at post, and 225 individuals (intervention group: *N* = 111; control group: *N* = 114) participated at follow-up. The sample included 87 men and 190 women aged 18–55 years at baseline (*M* = 34.41, *SD* = 10.66). In the intervention group, 105 of 139 participants (75.5%) completed AR training with *M* = 7.82 sessions attended (range 4 to 10). Only 18 (13.0%) did not attend any course session, and 16 (11.5%) terminated the training early after attending *M* = 3.25 sessions (range 1 to 6). More detailed information, including a CONSORT flow chart as well as sociodemographic and clinical characteristics of the sample, has been published elsewhere [45].

Of the total sample, 275 individuals (intervention group: *N* = 139; control group: *N* = 136) provided EMA data at baseline, 225 individuals (intervention group: *N* = 107; control group: *N* = 118) provided EMA data at post, and 142 individuals (intervention group: *N* = 66; control group: *N* = 76) provided EMA data at follow-up. A total of 277 subjects answered the EMA at baseline and/or post, 233 at post and/or follow-up, and 275 at baseline and/or follow-up. Thus, the analyses on changes from baseline to post, from post to follow-up, and from baseline to follow-up are based on 277, 233, and 275 individuals, respectively. The numbers of individuals and observations for individual outcomes at baseline, post, and follow-up are shown in Table 1 (symptom outcomes) and S1 Table (other psychological outcomes).

**Table 1:** Number of individuals and observations for each symptom outcome at baseline, post, and follow-up as well as from baseline to post, from post to follow-up, and from baseline to follow-up.

	Baseline (N = 275)		Post (N = 225)		Follow-up (N = 142)		Baseline to post (N = 277^1^)		Post to follow-up (N = 233^2^)		Baseline to follow-up (N = 275^3^)	
	Persons	Observ.	Persons	Observ.	Persons	Observ.	Persons	Observ.	Persons	Observ.	Persons	Observ.
Symptom outcome	N	N	N	N	N	N	N	N	N	N	N	N
DASS-total	275	1,684	225	1,326	141	817	277	3,010	233	2,143	275	2,501
DASS-depression	275	1,684	225	1,326	141	817	277	3,010	233	2,143	275	2,501
DASS-anxiety	275	1,684	225	1,326	141	817	277	3,010	233	2,143	275	2,501
DASS-stress	275	1,684	225	1,326	141	817	277	3,010	233	2,143	275	2,501
PROMIS-depression	275	8,126	225	6,421	142	3,987	277	14,547	233	10,408	275	12,113
PROMIS-anxiety	275	8,130	225	6,427	142	3,991	277	14,557	233	10,418	275	12,121
PROMIS-anger	275	8,128	225	6,425	142	3,987	277	14,553	233	10,412	275	12,115
PHQ-15-somatic symptoms	275	8,127	225	6,423	142	3,987	277	14,550	233	10,410	275	12,114
PROMIS-sleep	273	1,611	224	1,293	140	799	277	2,904	233	2,092	274	2,410

*Note*. The number of observations is lower for some outcomes because they were assessed only in the evening or under certain circumstances. (see methods section). Observ. = Observations. ^1^ Participants with EMA data at baseline and/or post. ^2^ Participants with EMA data at post and/or follow-up. ^3^ Participants with EMA data at baseline and/or follow-up.

As indicated by little’s MCAR test, the data were not completely missing at random (*p* < .001). Chi-squared tests showed that missing values for individual outcomes did not vary by sex or group (all *p*-values > .05). The only exception was that information on PROMIS-depression and positive affect was missing more often in the control group than in the intervention group, and that information on favorable and unfavorable coping as well as positive affect was missing more often in men than in women.

Sex and age differences in baseline outcome measures are shown in S2 Table. Compared with men, women had higher DASS-total scores (*β* = 0.13), DASS-stress scores (*β* = 0.15), PHQ-15-somatic symptoms (*β* = 0.31), and PROMIS-sleep problems (*β* = 0.18) but lower PROMIS-depression scores (*β* = -0.10). In addition, women reported lower positive affect (*β* = -0.05) but higher internal control beliefs (*β* = 0.14) as well as more favorable (*β* = 0.21) and more unfavorable (*β* = 0.38) coping.

Compared with younger individuals, older individuals had higher PROMIS-anger scores (*β* = 0.03 per decade), PHQ-15-somatic symptoms (*β* = 0.11), and PROMIS-sleep problems (*β* = 0.09) but lower PROMIS-depression scores (*β* = -0.04). In addition, older individuals reported lower internal (*β* = -0.07) and higher external (*β* = 0.08) control beliefs, higher self-efficacy (*β* = 0.05), and less unfavorable coping (*β* = -0.15).

### Intervention effects

Means and standard deviations for individual outcomes at baseline, post, and follow-up in the intervention and control group are shown in S3 Table. From baseline to post, all psychopathological symptoms decreased more in the intervention group than in the control group (time*group: range *β* = -0.31 for DASS-depression to *β* = -0.06 for PROMIS-anger; Table 2). From post to follow-up, psychopathological symptoms continued to decrease in both groups. However, this decrease tended to be larger in the control group than in the intervention group, so that only the intervention effects for PROMIS-depression (*β* = -0.10) and PROMIS-anger (*β* = -0.09) were maintained from baseline to follow-up.

**Table 2:** Symptom changes from baseline to post, from post to follow-up, and from baseline to follow-up in the intervention vs. control group (interactive effects: Group*time).

	From baseline to post (N = 277^1^)					From post to follow-up (N = 233^2^)					From baseline to follow-up (N = 275^3^)				
	Group*time					Group*time					Group*time				
Symptom outcome	β	95% CI		p_raw_	p_cor_	β	95% CI		p_raw_	p_cor_	β	95% CI		p_raw_	p_cor_
DASS-total	-0.27	-0.38	-0.17	< .001	< .001	0.22	0.09	0.36	.001	.005	0.01	-0.12	0.15	.855	.855
DASS-depression	-0.31	-0.42	-0.20	< .001	< .001	0.13	0.00	0.27	.049	.110	-0.14	-0.27	0.00	.049	.147
DASS-anxiety	-0.19	-0.30	-0.09	< .001	< .001	0.09	-0.04	0.22	.167	.188	0.02	-0.11	0.15	.750	.844
DASS-stress	-0.19	-0.31	-0.08	.001	.001	0.26	0.11	0.40	< .001	< .001	0.09	-0.05	0.24	.207	.407
PROMIS-depression	-0.19	-0.24	-0.14	< .001	< .001	0.09	0.03	0.15	.004	.012	-0.10	-0.16	-0.04	.001	.009
PROMIS-anxiety	-0.11	-0.16	-0.05	< .001	< .001	0.06	-0.01	0.13	.085	.149	-0.04	-0.11	0.03	.226	.407
PROMIS-anger	-0.06	-0.12	0.00	.034	.034	-0.06	-0.13	0.01	.099	.149	-0.09	-0.16	-0.02	.009	.041
PHQ-15-somatic symptoms	-0.07	-0.12	-0.02	.005	.006	0.01	-0.06	0.07	.872	.872	-0.02	-0.09	0.04	.448	.672
PROMIS-sleep	-0.20	-0.32	-0.08	.001	.001	0.11	-0.04	0.26	.142	.183	-0.05	-0.19	0.10	.527	.678

*Note*. β = standardized beta-coefficient from multilevel mixed-effects linear regression models, adjusted for sex and age. CI = confidence interval. p_raw_ = uncorrected p-value. p_cor_ = corrected p-value using the Benjamini-Hochberg procedure. All outcomes were log-transformed and standardized across all waves based on the pooled standard deviation of the intervention and control group at baseline. ^1^ Participants with EMA data at baseline and/or post. ^2^ Participants with EMA data at post and/or follow-up. ^3^ Participants with EMA data at baseline and/or follow-up. The exact number of participants and observations per outcome and model is shown in Table 1.

Intervention effects for other psychological outcomes are shown in S4 Table. From baseline to post, all other emotional (positive affect: *β* = 0.19), cognitive (internal control beliefs: *β* = 0.15), and behavioral (favorable coping: *β* = 0.60; unfavorable coping: *β* = -0.41) outcomes improved more in the intervention group than in the control group, except for external control beliefs and self-efficacy. The effects from baseline to follow-up were similar. In addition, self-efficacy increased more in the intervention than in the control group from baseline to follow-up (*β* = 0.19).

At each EMA, participants were asked to indicate how many times they had consciously attempted to relax since the last assessment. (Two observations were excluded from these analyses because an unrealistically high number of 88 and 102 relaxation attempts, respectively, was reported since the last assessment.) The number of relaxation attempts did not differ between the two groups at baseline (intervention: *M* = 0.15, *SD* = 0.50; control: *M* = 0.16, *SD* = 0.49; *t*(8120) = 1.01, *p* = .312). At post, the intervention group (*M* = 0.97, *SD* = 1.60) attempted to consciously relax more often than the control group (*M* = 0.06, *SD* = 0.29; *t*(6413) = -32.47, *p* < .001). Although the number of relaxations attempts in the intervention group decreased from post to follow-up, this group difference was still significant at follow-up (intervention: *M* = 0.35, *SD* = 0.83; control: *M* = 0.05, *SD* = 0.25; *t*(3982) = -15.96, *p* < .001).

### The role of sex

From baseline to post, the intervention effects for PROMIS-depression (time*group*sex: *β* = -0.16) and PHQ-15-somatic symptoms (*β* = -0.39) were stronger in women than in men (Table 3). From baseline to follow-up, the intervention effects for PROMIS-anxiety (*β* = -0.26) and PHQ-15-somatic symptoms (*β* = -0.27) were stronger in women than in men.

**Table 3:** Sex differences with respect to symptom changes from baseline to post, from post to follow-up, and from baseline to follow-up in the intervention vs. control group (interactive effects: Group*time*female sex).

	From baseline to post (N = 277^1^)					From post to follow-up (N = 233^2^)					From baseline to follow-up (N = 275^3^)				
	Group*time*female sex					Group*time*female sex					Group*time*female sex				
Symptom outcome	β	95% CI		p_raw_	p_cor_	β	95% CI		p_raw_	p_cor_	β	95% CI		p_raw_	p_cor_
DASS-total	-0.13	-0.37	0.10	.267	.393	0.09	-0.20	0.38	.550	.707	-0.30	-0.59	-0.01	.043	.106
DASS-depression	-0.16	-0.40	0.08	.198	.356	0.17	-0.12	0.46	.257	.583	-0.29	-0.58	0.00	.047	.106
DASS-anxiety	-0.16	-0.39	0.07	.178	.356	0.00	-0.27	0.28	.976	.976	-0.22	-0.49	0.06	.124	.159
DASS-stress	-0.09	-0.34	0.16	.496	.510	0.02	-0.29	0.33	.905	.976	-0.23	-0.54	0.08	.147	.165
PROMIS-depression	-0.16	-0.26	-0.05	.004	.018	0.16	0.03	0.29	.015	.068	-0.11	-0.23	0.02	.095	.159
PROMIS-anxiety	-0.04	-0.16	0.08	.510	.510	-0.08	-0.22	0.07	.290	.583	-0.26	-0.40	-0.12	< .001	< .001
PROMIS-anger	-0.11	-0.23	0.01	.083	.249	0.08	-0.08	0.23	.324	.583	-0.12	-0.27	0.03	.120	.159
PHQ-15-somatic symptoms	-0.39	-0.50	-0.28	< .001	< .001	0.26	0.13	0.39	< .001	< .001	-0.27	-0.40	-0.14	< .001	< .001
PROMIS-sleep	-0.13	-0.39	0.12	.306	.393	0.11	-0.21	0.43	.502	.707	-0.15	-0.46	0.16	.339	.339

*Note*. β = standardized beta-coefficient from multilevel mixed-effects linear regression models, adjusted for age. CI = confidence interval. p_raw_ = uncorrected p-value. p_cor_ = corrected p-value using the Benjamini-Hochberg procedure. All outcomes were log-transformed and standardized across all waves based on the pooled standard deviation of the intervention and control group at baseline. ^1^ Participants with EMA data at baseline and/or post. ^2^ Participants with EMA data at post and/or follow-up. ^3^ Participants with EMA data at baseline and/or follow-up. The exact number of participants and observations per outcome and model is shown in Table 1.

From baseline to post, the intervention effects for positive affect (*β* = 0.22), self-efficacy (*β* = 0.35), and favorable coping (*β* = 0.79) were stronger in women, whereas the intervention effect for unfavorable coping was stronger in men (*β* = 0.75; S5 Table). From baseline to follow-up, the intervention effect for external control beliefs was stronger in men (*β* = 0.34), whereas the intervention effect for favorable coping was stronger in women (*β* = 0.96).

Overall, AR training tended to have more favorable effects on mental health in women. However, the intervention efficacy for certain outcomes (i.e., external control beliefs and unfavorable coping) was higher in men.

### The role of age

Especially in the long term, AR tended to be more effective in older individuals (Table 4): The intervention effects (a) for PHQ-15-somatic symptoms from baseline to post and (b) for DASS-anxiety, PROMIS-depression, PROMIS-anxiety, PROMIS-anger, and PHQ-15-somatic symptoms from baseline to follow-up were stronger in older (vs. younger) individuals (time*group*age: range *β* = -0.27 to *β* = -0.08 per 10 years older).

**Table 4:** Age differences with respect to symptom changes from baseline to post, from post to follow-up, and from baseline to follow-up in the intervention vs. control group (interactive effects: Group*time*age).

	From baseline to post (N = 277^1^)					From post to follow-up (N = 233^2^)					From baseline to follow-up (N = 275^3^)				
	Group*time*age					Group*time*age					Group*time*age				
Symptom outcome	β	95% CI		p_raw_	p_cor_	β	95% CI		p_raw_	p_cor_	β	95% CI		p_raw_	p_cor_
DASS-total	-0.08	-0.18	0.03	.145	.326	-0.02	-0.15	0.11	.781	.925	-0.10	-0.23	0.02	.106	.159
DASS-depression	-0.03	-0.13	0.08	.635	.673	0.01	-0.12	0.13	.925	.925	-0.03	-0.16	0.10	.642	.722
DASS-anxiety	-0.02	-0.12	0.08	.673	.673	-0.13	-0.25	-0.01	.028	.252	-0.27	-0.39	-0.16	< .001	< .001
DASS-stress	-0.13	-0.24	-0.02	.025	.113	0.02	-0.12	0.15	.807	.925	-0.09	-0.22	0.05	.208	.267
PROMIS-depression	-0.01	-0.06	0.03	.618	.673	-0.02	-0.07	0.04	.573	.925	-0.08	-0.13	-0.02	.005	.009
PROMIS-anxiety	-0.05	-0.11	0.00	.038	.114	0.01	-0.06	0.07	.870	.925	-0.11	-0.17	-0.04	.001	.002
PROMIS-anger	-0.04	-0.09	0.02	.194	.349	-0.06	-0.12	0.01	.102	.459	-0.11	-0.17	-0.04	.001	.002
PHQ-15-somatic symptoms	-0.21	-0.25	-0.16	< .001	< .001	0.04	-0.02	0.09	.210	.630	-0.14	-0.20	-0.09	< .001	< .001
PROMIS-sleep	0.04	-0.08	0.15	.526	.673	-0.02	-0.15	0.12	.812	.925	0.02	-0.11	0.16	.744	.744

*Note*. β = standardized beta-coefficient from multilevel mixed-effects linear regression models, adjusted for sex. CI = confidence interval. p_raw_ = uncorrected p-value. p_cor_ = corrected p-value using the Benjamini-Hochberg procedure. All outcomes were log-transformed and standardized across all waves based on the pooled standard deviation of the intervention and control group at baseline. The age variable (in years) was divided by 10 to ensure that the effects did not become too small to be presented rounded. ^1^ Participants with EMA data at baseline and/or post. ^2^ Participants with EMA data at post and/or follow-up. ^3^ Participants with EMA data at baseline and/or follow-up. The exact number of participants and observations per outcome and model is shown in Table 1.

Fewer interactions with age were found for other psychological outcomes (S6 Table): From baseline to follow-up, the intervention efficacy for internal control beliefs was stronger in older individuals (*β* = 0.20 per 10 years older), whereas the intervention efficacy for favorable coping was stronger in younger individuals (*β* = -0.45 per 10 years older).

### The role of initial symptom severity

As shown in Table 5, individuals with more severe baseline symptoms tended to benefit more from AR training: Except for DASS-anxiety, PROMIS-anger, and PROMIS-sleep, the intervention effects for all symptoms from baseline to post were stronger among individuals with higher initial levels of the respective symptoms (time*group*symptom severity at baseline: range *β* = -0.34 for PROMIS-depression to *β* = -0.16 for DASS-depression). However, the intervention effect for PROMIS-sleep was stronger among participants with fewer sleep problems at baseline (*β* = 0.25).

**Table 5:** Differences with respect to symptom changes from baseline to post, from post to follow-up, and from baseline to follow-up by baseline symptom severity in the intervention vs. control group (interactive effects: Group*time*baseline symptom severity).

	From baseline to post (N = 277^1^)					From post to follow-up (N = 233^2^)					From baseline to follow-up (N = 275^3^)				
	Group*time*baseline symptom severity					Group*time*baseline symptom severity					Group*time*baseline symptom severity				
Symptom outcome	β	95% CI		p_raw_	p_cor_	β	95% CI		p_raw_	p_cor_	β	95% CI		p_raw_	p_cor_
DASS-total	-0.21	-0.35	-0.08	.002	.003	0.07	-0.10	0.24	.422	.760	-0.17	-0.33	-0.02	.029	.044
DASS-depression	-0.16	-0.30	-0.03	.015	.019	0.01	-0.16	0.18	.925	.925	-0.13	-0.29	0.02	.094	.115
DASS-anxiety	-0.02	-0.15	0.10	.715	.715	0.18	0.03	0.34	.020	.060	0.16	0.02	0.30	.023	.041
DASS-stress	-0.26	-0.41	-0.11	.001	.002	0.05	-0.15	0.24	.641	.824	-0.26	-0.43	-0.08	.004	.012
PROMIS-depression	-0.34	-0.41	-0.28	< .001	< .001	0.15	0.08	0.23	< .001	< .001	-0.12	-0.20	-0.05	.001	.005
PROMIS-anxiety	-0.22	-0.30	-0.14	< .001	< .001	0.02	-0.09	0.12	.753	.847	-0.20	-0.30	-0.10	< .001	< .001
PROMIS-anger	-0.06	-0.16	0.03	.183	.206	0.10	-0.01	0.22	.081	.182	0.14	0.03	0.25	.014	.032
PHQ-15-somatic symptoms	-0.20	-0.27	-0.14	< .001	< .001	0.24	0.15	0.32	< .001	< .001	0.07	-0.01	0.15	.102	.115
PROMIS-sleep	0.25	0.10	0.40	.001	.002	-0.06	-0.25	0.13	.516	.774	0.13	-0.03	0.29	.122	.122

*Note*. β = standardized beta-coefficient from multilevel mixed-effects linear regression models, adjusted for sex and age. CI = confidence interval. p_raw_ = uncorrected p-value. p_cor_ = corrected p-value using the Benjamini-Hochberg procedure. All outcomes were log-transformed and standardized across all waves based on the pooled standard deviation of the intervention and control group at baseline. ^1^ Participants with EMA data at baseline and/or post. ^2^ Participants with EMA data at post and/or follow-up. ^3^ Participants with EMA data at baseline and/or follow-up. The exact number of participants and observations per outcome and model is shown in Table 1.

From baseline to follow-up, the intervention effects for DASS-total (*β* = -0.17), DASS-stress (*β* = -0.26), PROMIS-depression (*β* = -0.12), and PROMIS-anxiety (*β* = -0.20) were also stronger among individuals with higher initial levels of the respective symptoms. Conversely, the intervention effects for DASS-anxiety (*β* = -0.17) and PROMIS-anger (*β* = -0.17) from baseline to follow-up were stronger among individuals with lower initial levels of the respective symptoms.

Regarding other psychological outcomes (S7 Table), the intervention efficacy for internal control beliefs from baseline to post (*β* = -0.24) and from baseline to follow-up (*β* = -0.42) was stronger among individuals with lower internal control beliefs at baseline.

## Discussion

This analysis of randomized controlled indicated prevention trial data in adults with elevated psychopathological symptoms but without full-threshold mental disorders at study entry examined whether group-based AR training effectively reduced psychopathological symptoms in real-life contexts, assessed in time to experience via smartphone-based EMA. Results illustrate that AR training led to significant improvements in all psychopathological symptoms examined and in health-related emotional, cognitive, and behavioral characteristics. A large proportion of these improvements were not only observed from baseline to post but persisted through 12-month follow-up.

Compared with the control group, the intervention group experienced greater decreases in several psychopathological symptoms from baseline to post, including depressive, anxiety, stress, anger, and somatic symptoms as well as sleep problems. Moreover, the intervention group improved more in terms of positive affect, perceived control, and coping behaviors due to AR training. A large proportion of these effects was maintained over the long term and still seen after one year. These results are consistent with our hypotheses and with previous evidence that AR was effective in reducing psychopathological symptoms (e.g., anxiety), particularly in patient samples [11–25]. Furthermore, our findings are consistent with previous findings from the same study that AR effectively reduced psychopathological symptoms assessed with conventional questionnaires (instead of EMA) [45].

### Intervention effects

The present findings not only replicate but substantially extend previous evidence on AR: Most prior studies focused on small samples, patients with full-threshold mental disorders, or single outcomes assessed with conventional questionnaires. In contrast, our study was based on a comparatively large sample of high-risk individuals with elevated psychopathological symptoms but without full-threshold mental disorders at study entry. Various internalizing and externalizing symptoms and other health-related psychological outcomes were assessed using established scales. To minimize retrospective memory and reporting biases and evaluate the efficacy in real-life contexts, diagnostic information was collected via smartphone-based EMA in time to experience five times daily for one week at each wave. Taken together, our findings suggest that AR training promotes successful transfer to everyday life, indicating that AR can be used effectively to manage daily stressful situations.

### The role of initial symptom severity

For several outcomes, AR tended to be more effective in individuals with higher baseline symptoms. This result is consistent with our hypotheses based on motivational theories [47,48]: Individuals with more severe mental health problems might be more motivated to learn AR. They might be more compliant during the intervention and use AR more frequently in daily life [26]. Moreover, individuals with more severe symptoms have more room for improvement, which could explain why they benefit more [67].

### The role of sex

AR tended to be more effective in women than in men, particularly with respect to internalizing outcomes such as anxiety and somatic symptoms. These results are in line with our hypotheses and with meta-analytic findings that relaxation training reduces anxiety more effectively in women [49]. However, for a few outcomes such as external control beliefs and unfavorable coping (i.e., flight and avoidance), the efficacy was higher in men. On average, women are more prone to internalizing symptoms, while men are more prone to externalizing symptoms [68,69]. Thus, one could speculate whether women are also more likely to experience changes in internalizing symptoms, while men are more likely to experience changes in externalizing symptoms due to AR. Additional research, however, is needed to substantiate these results for individual symptom outcomes.

### The role of age

It was found that AR tended to be more effective in older individuals. This result is inconsistent with our hypotheses and with meta-analytic evidence that relaxation training was less effective in older individuals [49]. Older individuals might benefit less from relaxation training due to greater difficulties understanding the introduction and practicing at home [49]. However, unlike many previous studies, our sample only included younger and middle-aged individuals (<55 years), which might explain inconsistent results. Middle-aged adults tend to be healthier and more flexible than older people as well as exposed to higher levels of strain and stressors than younger people (i.e., due to work-family conflicts) [70]. Therefore, they might benefit more from AR training, which could explain our results.

### Limitations

Our study is not without limitations: Only short scales were used in the EMA, which may be less reliable than other, more comprehensive questionnaires. Diagnostic information was collected via self-report, which may differ from clinical expert ratings or behavioral observations.

There was no active control condition, so it was not possible to test whether AR or unspecific factors such as treatment expectations and regular contact with psychological trainers led to symptom improvements. However, compared with the control group, participants in the intervention group were more likely to consciously relax in daily life at post and follow-up, suggesting that the intervention led to behavioral changes that explain the intervention efficacy.

Only one follow-up was conducted at 12 months, so nuanced symptom changes in the months after the intervention could not be examined. Additional follow-ups are also needed to evaluate the long-term efficacy after more than one year.

The study was based on a convenient sample of adults aged between 18 and 55 and living in the Dresden area. Therefore, the present results may not be fully generalizable to other age and regional groups. Moreover, more women (68.6%) than men participated in the study, and many individuals (48.7%) did not provide EMA data at 12-month follow-up. At the same time, the intervention efficacy tended to be higher in women and individuals with more severe baseline symptoms, and more burdened individuals might have been more likely to skip the EMA at follow-up. Thus, one could speculate whether the intervention effects might have been overestimated. In this study, participants were given a smartphone to answer the EMA. In future studies, it may be useful to enable participants to conduct the EMA on their own smartphone to reduce the risk of dropout.

## Conclusions

Our study suggests that group-based AR training significantly reduces psychopathological symptoms in daily life and leads to improvements in health-related emotional, cognitive, and behavioral traits. These favorable effects of AR training tend to be stronger in women than in men, in older than in younger individuals, and in participants with higher vs. lower baseline symptoms.

Our findings underscore the importance of promoting transfer from the training setting to everyday life in prevention and early intervention programs. Future research may investigate (a) which contextual and situational factors increase or decrease the efficacy of AR in everyday life and (b) how to improve the long-term efficacy of AR training. In the intervention group, the number of relaxation attempts decreased from post to follow-up, highlighting the importance of additional booster sessions (e.g., 3 or 6 months after AR training). In this context, mobile technologies could be useful to remind participants regularly to use AR in stressful daily situations. Furthermore, the effectiveness in everyday life could be tested among particularly stressed groups such as health professionals, shift workers, or single parents.

## Supporting information

S1 Checklist CONSORT 2010 checklist of information to include when reporting a randomised trial*. (DOC)

S1 Table Number of individuals and observations for other psychological outcomes at baseline, post, and follow-up as well as from baseline to post, from post to follow-up, and from baseline to follow-up. (DOCX)

S2 Table Associations of sex and age with individual outcome measures at baseline (N = 275). (DOCX)

S3 Table Means and standard deviations for other psychological outcomes at baseline, post, and follow-up in the intervention (N = 139) and control (N = 138) group (total: N = 277). (DOCX)

S4 Table Changes in other psychological outcomes from baseline to post, from post to follow-up, and from baseline to follow-up in the intervention vs. control group (interactive effects: Group * time). (DOCX)

S5 Table Sex differences with respect to changes in other psychological outcomes from baseline to post, from post to follow-up, and from baseline to follow-up in the intervention vs. control group (interactive effects: Group * time * female sex). (DOCX)

S6 Table Age differences with respect to changes in other psychological outcomes from baseline to post, from post to follow-up, and from baseline to follow-up in the intervention vs. control group (interactive effects: Group * time * age). (DOCX)

S7 Table Differences with respect to changes in other psychological outcomes from baseline to post, from post to follow-up, and from baseline to follow-up by baseline levels of the respective outcome in the intervention vs. control group (interactive effects: Group * time * baseline symptom severity). (DOCX)
