# Supplementary material for: Ecological momentary assessment and applied relaxation: Results of a randomized indicated preventive trial in individuals at increased risk for mental disorders
Source: PLoS One. 2023 Jun 8;18(6):e0286750. doi: 10.1371/journal.pone.0286750 (PMC10249886; doi:10.1371/journal.pone.0286750)
Supplement: S1 Table — (DOCX) [file pone.0286750.s002.docx]

Table S1

*Number of individuals and observations for other psychological outcomes at baseline, post, and follow-up as well as from baseline to post, from post to follow-up, and from baseline to follow-up*

|  | Baseline  (N = 275) | | Post  (N = 225) | | Follow-up  (N = 142) | | Baseline to post  (N = 277^1^) | | Post to follow-up  (N = 233^2^) | | Baseline to follow-up  (N = 275^3^) | |
| --- | --- | --- | --- | --- | --- | --- | --- | --- | --- | --- | --- | --- |
|  | Persons | Observ. | Persons | Observ. | Persons | Observ. | Persons | Observ. | Persons | Observ. | Persons | Observ. |
| Outcome | N | N | N | N | N | N | N | N | N | N | N | N |
| Positive affect | 275 | 8,126 | 225 | 6,417 | 142 | 3,984 | 277 | 14,543 | 233 | 10,401 | 275 | 12,110 |
| Internal control beliefs | 275 | 1,683 | 225 | 1,324 | 141 | 817 | 277 | 3,007 | 233 | 2,141 | 275 | 2,500 |
| External control beliefs | 275 | 1,683 | 225 | 1,324 | 141 | 817 | 277 | 3,007 | 233 | 2,141 | 275 | 2,500 |
| Self-efficacy | 275 | 1,683 | 225 | 1,324 | 141 | 817 | 277 | 3,007 | 233 | 2,141 | 275 | 2,500 |
| Favorable coping | 226 | 545 | 166 | 374 | 87 | 164 | 248 | 919 | 187 | 538 | 238 | 709 |
| Unfavorable coping | 224 | 545 | 166 | 373 | 87 | 164 | 247 | 918 | 187 | 537 | 236 | 709 |

*Note.* The number of observations is lower for some outcomes because they were assessed only in the evening or under certain circumstances (see methods section). Observ. = Observations. ^1^ Participants with EMA data at baseline and/or post. ^2^ Participants with EMA data at post and/or follow-up. ^3^ Participants with EMA data at baseline and/or follow-up.
