# Supplementary material for: Ecological momentary assessment and applied relaxation: Results of a randomized indicated preventive trial in individuals at increased risk for mental disorders
Source: PLoS One. 2023 Jun 8;18(6):e0286750. doi: 10.1371/journal.pone.0286750 (PMC10249886; doi:10.1371/journal.pone.0286750)
Supplement: S2 Table — (DOCX) [file pone.0286750.s003.docx]

Table S2

*Associations of sex and age with individual outcome measures at baseline (N = 275^1^)*

|  | Sex^2^ | | | | Age^3^ | | | |
| --- | --- | --- | --- | --- | --- | --- | --- | --- |
| Outcome | β | 95% CI | | p | β | 95% CI | | p |
| DASS-total | 0.13 | 0.02 | 0.23 | .018 | -0.03 | -0.07 | 0.02 | .215 |
| DASS-depression | 0.05 | -0.06 | 0.15 | .364 | -0.02 | -0.06 | 0.03 | .394 |
| DASS-anxiety | 0.09 | -0.01 | 0.20 | .080 | -0.04 | -0.09 | 0.00 | .076 |
| DASS-stress | 0.15 | 0.05 | 0.26 | .004 | -0.01 | -0.05 | 0.04 | .705 |
| PROMIS-depression | -0.10 | -0.14 | -0.05 | <.001 | -0.04 | -0.06 | -0.02 | .001 |
| PROMIS-anxiety | -0.01 | -0.06 | 0.04 | .659 | -0.02 | -0.04 | 0.00 | .095 |
| PROMIS-anger | -0.01 | -0.06 | 0.04 | .667 | 0.03 | 0.01 | 0.05 | .003 |
| PHQ-15-somatic symptoms | 0.31 | 0.26 | 0.35 | <.001 | 0.11 | 0.09 | 0.13 | <.001 |
| PROMIS-sleep | 0.18 | 0.07 | 0.28 | .001 | 0.09 | 0.04 | 0.13 | <.001 |
|  |  |  |  |  |  |  |  |  |
| Positive affect | -0.05 | -0.10 | 0.00 | .048 | -0.01 | -0.03 | 0.01 | .259 |
| Internal control beliefs | 0.14 | 0.03 | 0.24 | .012 | -0.07 | -0.11 | -0.03 | .002 |
| External control beliefs | -0.02 | -0.13 | 0.08 | .673 | 0.08 | 0.03 | 0.12 | .001 |
| Self-efficacy | 0.08 | -0.02 | 0.19 | .128 | 0.05 | 0.00 | 0.09 | .041 |
| Favorable coping | 0.21 | 0.02 | 0.40 | .031 | 0.01 | -0.08 | 0.09 | .900 |
| Unfavorable coping | 0.38 | 0.19 | 0.56 | <.001 | -0.15 | -0.23 | -0.07 | <.001 |

*Note.* β = standardized beta-coefficient from linear regressions. CI = confidence interval. p = p-value. All outcomes were log-transformed and standardized across all waves based on the pooled standard deviation of the intervention and control group at baseline. The age variable was divided by 10 to ensure that the effects did not become too small to be presented rounded. ^1^ The exact numbers of participants and observations per outcome and model are shown in Table 1 and Table S1. ^2^ Adjusted for age. ^3^ Adjusted for sex.
