# Supplementary material for: Ecological momentary assessment and applied relaxation: Results of a randomized indicated preventive trial in individuals at increased risk for mental disorders
Source: PLoS One. 2023 Jun 8;18(6):e0286750. doi: 10.1371/journal.pone.0286750 (PMC10249886; doi:10.1371/journal.pone.0286750)
Supplement: S3 Table — (DOCX) [file pone.0286750.s004.docx]

Table S3

*Means and standard deviations for other psychological outcomes at baseline, post, and follow-up in the intervention (N = 139) and control (N = 138) group (total: N = 277^1^)*

|  | Baseline (N = 275^1^) | | | | Post (N = 225^1^) | | | | Follow-up (N = 142^1^) | | | |
| --- | --- | --- | --- | --- | --- | --- | --- | --- | --- | --- | --- | --- |
|  | Intervention group  (N = 139) | | Control group  (N = 136) | | Intervention group  (N = 107) | | Control group  (N = 118) | | Intervention group  (N = 66) | | Control group  (N = 76) | |
| Outcome | M | SD | M | SD | M | SD | M | SD | M | SD | M | SD |
| DASS-total | 7.31 | 7.40 | 8.56 | 9.08 | 5.49 | 7.27 | 8.50 | 9.84 | 4.55 | 5.37 | 5.22 | 7.22 |
| DASS-depression | 6.53 | 8.49 | 7.98 | 10.39 | 4.80 | 7.93 | 8.86 | 11.76 | 3.96 | 5.45 | 4.88 | 7.78 |
| DASS-anxiety | 4.19 | 5.59 | 4.28 | 6.36 | 3.39 | 5.91 | 4.24 | 7.14 | 2.85 | 4.68 | 2.52 | 4.04 |
| DASS-stress | 11.22 | 11.89 | 13.43 | 14.39 | 8.29 | 10.94 | 12.39 | 14.31 | 6.84 | 8.61 | 8.28 | 12.41 |
| PROMIS-depression | 4.34 | 7.95 | 5.24 | 8.97 | 3.39 | 6.64 | 6.34 | 10.23 | 2.61 | 4.21 | 3.37 | 6.89 |
| PROMIS-anxiety | 7.42 | 8.82 | 8.28 | 9.91 | 6.27 | 8.07 | 8.00 | 10.30 | 5.32 | 6.63 | 5.69 | 8.08 |
| PROMIS-anger | 6.40 | 9.49 | 7.21 | 10.14 | 5.81 | 9.13 | 6.96 | 10.44 | 4.76 | 7.39 | 4.92 | 7.81 |
| PHQ-15-somatic symptoms | 7.46 | 7.89 | 7.05 | 6.72 | 6.20 | 6.78 | 6.98 | 7.57 | 5.62 | 6.13 | 5.39 | 5.50 |
| PROMIS-sleep | 26.20 | 17.24 | 26.81 | 18.36 | 24.08 | 16.32 | 27.98 | 18.14 | 22.44 | 16.05 | 24.14 | 17.02 |
|  |  |  |  |  |  |  |  |  |  |  |  |  |
| Positive affect | 40.99 | 22.10 | 40.78 | 21.70 | 49.31 | 24.57 | 42.19 | 22.23 | 49.09 | 23.56 | 43.24 | 22.03 |
| Internal control beliefs | 74.32 | 18.86 | 72.10 | 19.88 | 75.89 | 19.06 | 73.33 | 21.47 | 78.30 | 17.23 | 73.45 | 21.23 |
| External control beliefs | 22.42 | 19.14 | 26.05 | 21.34 | 22.61 | 21.17 | 26.29 | 23.85 | 20.82 | 19.30 | 24.00 | 21.90 |
| Self-efficacy | 69.08 | 19.89 | 67.90 | 21.60 | 73.06 | 19.40 | 68.76 | 21.23 | 75.67 | 18.02 | 71.48 | 20.08 |
| Favorable coping | 36.83 | 19.85 | 36.32 | 18.30 | 52.46 | 24.27 | 36.15 | 18.71 | 46.10 | 22.87 | 29.24 | 17.73 |
| Unfavorable coping | 30.89 | 19.32 | 27.29 | 19.30 | 24.37 | 17.73 | 29.16 | 19.11 | 21.79 | 17.35 | 25.35 | 18.24 |

*Note.* M = mean. SD = standard deviation. ^1^ Two individuals provided EMA data at post but not at baseline. ^1^ The exact numbers of participants and observations per outcome are shown in Table 1 and Table S1.
