# Supplementary material for: Ecological momentary assessment and applied relaxation: Results of a randomized indicated preventive trial in individuals at increased risk for mental disorders
Source: PLoS One. 2023 Jun 8;18(6):e0286750. doi: 10.1371/journal.pone.0286750 (PMC10249886; doi:10.1371/journal.pone.0286750)
Supplement: S4 Table — (DOCX) [file pone.0286750.s005.docx]

Table S4

*Changes in other psychological outcomes from baseline to post, from post to follow-up, and from baseline to follow-up in the intervention vs. control group (interactive effects: group * time)*

|  | From baseline to post (N = 277^1^) | | | | | From post to follow-up (N = 233^2^) | | | | | From baseline to follow-up (N = 275^3^) | | | | |
| --- | --- | --- | --- | --- | --- | --- | --- | --- | --- | --- | --- | --- | --- | --- | --- |
|  | Group * time | | | | | Group * time | | | | | Group * time | | | | |
| Outcome | β | 95% CI | | p_raw_ | p_cor_ | β | 95% CI | | p_raw_ | p_cor_ | β | 95% CI | | p_raw_ | p_cor_ |
| Positive affect | 0.19 | 0.13 | 0.25 | <.001 | <.001 | 0.00 | -0.08 | 0.07 | .961 | .961 | 0.14 | 0.07 | 0.21 | <.001 | <.001 |
| Internal control beliefs | 0.15 | 0.01 | 0.29 | .032 | .048 | 0.25 | 0.05 | 0.44 | .012 | .072 | 0.33 | 0.17 | 0.48 | <.001 | <.001 |
| External control beliefs | -0.07 | -0.16 | 0.03 | .170 | .170 | -0.03 | -0.15 | 0.09 | .634 | .951 | -0.11 | -0.23 | 0.01 | .068 | .068 |
| Self-efficacy | 0.12 | -0.01 | 0.24 | .064 | .077 | 0.15 | 0.00 | 0.29 | .056 | .168 | 0.19 | 0.05 | 0.33 | .010 | .012 |
| Favorable coping | 0.60 | 0.40 | 0.81 | <.001 | <.001 | 0.09 | -0.21 | 0.40 | .547 | .951 | 0.74 | 0.43 | 1.06 | <.001 | <.001 |
| Unfavorable coping | -0.41 | -0.64 | -0.19 | <.001 | <.001 | 0.01 | -0.35 | 0.37 | .957 | .961 | -0.54 | -0.85 | -0.22 | .001 | .002 |

*Note.* β = standardized beta-coefficient from multilevel mixed-effects linear regressions, adjusted for sex and age. CI = confidence interval. p_raw_ = uncorrected p-value. p_cor_ = corrected p-value using the Benjamini-Hochberg procedure. All outcomes were log-transformed and standardized across all waves based on the pooled standard deviation of the intervention and control group at baseline. ^1^ Participants with EMA data at baseline and/or post. ^2^ Participants with EMA data at post and/or follow-up. ^3^ Participants with EMA data at baseline and/or follow-up. The exact number of participants and observations per outcome and model is shown in Table S1.
