# Supplementary material for: Ecological momentary assessment and applied relaxation: Results of a randomized indicated preventive trial in individuals at increased risk for mental disorders
Source: PLoS One. 2023 Jun 8;18(6):e0286750. doi: 10.1371/journal.pone.0286750 (PMC10249886; doi:10.1371/journal.pone.0286750)
Supplement: S5 Table — (DOCX) [file pone.0286750.s006.docx]

Table S5

*Sex differences with respect to changes in other psychological outcomes from baseline to post, from post to follow-up, and from baseline to follow-up in the intervention vs. control group (interactive effects: group * time * female sex)*

|  | From baseline to post (N = 277^1^) | | | | | From post to follow-up (N = 233^2^) | | | | | From baseline to follow-up (N = 275^3^) | | | | |
| --- | --- | --- | --- | --- | --- | --- | --- | --- | --- | --- | --- | --- | --- | --- | --- |
|  | Group * time * female sex | | | | | Group * time * female sex | | | | | Group * time * female sex | | | | |
| Outcome | β | 95% CI | | p_raw_ | p_cor_ | β | 95% CI | | p_raw_ | p_cor_ | β | 95% CI | | p_raw_ | p_cor_ |
| Positive affect | 0.22 | 0.09 | 0.35 | .001 | .003 | -0.17 | -0.33 | -0.01 | .037 | .074 | 0.14 | -0.01 | 0.30 | .072 | .144 |
| Internal control beliefs | 0.16 | -0.14 | 0.46 | .306 | .306 | -0.25 | -0.67 | 0.17 | .244 | .328 | -0.19 | -0.53 | 0.15 | .271 | .325 |
| External control beliefs | 0.14 | -0.07 | 0.34 | .200 | .240 | 0.14 | -0.11 | 0.40 | .273 | .328 | 0.34 | 0.09 | 0.60 | .008 | .024 |
| Self-efficacy | 0.35 | 0.08 | 0.63 | .012 | .018 | -0.35 | -0.67 | -0.03 | .034 | .074 | 0.20 | -0.11 | 0.50 | .202 | .303 |
| Favorable coping | 0.79 | 0.32 | 1.27 | .001 | .003 | -0.21 | -0.90 | 0.48 | .547 | .547 | 0.96 | 0.25 | 1.67 | .008 | .024 |
| Unfavorable coping | 0.75 | 0.23 | 1.28 | .005 | .010 | -1.26 | -2.05 | -0.46 | .002 | .012 | -0.34 | -1.04 | 0.36 | .344 | .344 |

*Note.* β = standardized beta-coefficient from multilevel mixed-effects linear regressions, adjusted for age. CI = confidence interval. p_raw_ = uncorrected p-value. p_cor_ = corrected p-value using the Benjamini-Hochberg procedure. All outcomes were log-transformed and standardized across all waves based on the pooled standard deviation of the intervention and control group at baseline. ^1^ Participants with EMA data at baseline and/or post. ^2^ Participants with EMA data at post and/or follow-up. ^3^ Participants with EMA data at baseline and/or follow-up. The exact number of participants and observations per outcome and model is shown in Table S1.
