# Supplementary material for: Ecological momentary assessment and applied relaxation: Results of a randomized indicated preventive trial in individuals at increased risk for mental disorders
Source: PLoS One. 2023 Jun 8;18(6):e0286750. doi: 10.1371/journal.pone.0286750 (PMC10249886; doi:10.1371/journal.pone.0286750)
Supplement: S6 Table — (DOCX) [file pone.0286750.s007.docx]

Table S6

*Age differences with respect to changes in other psychological outcomes from baseline to post, from post to follow-up, and from baseline to follow-up in the intervention vs. control group (interactive effects: group * time * age)*

|  | From baseline to post (N = 277^1^) | | | | | From post to follow-up (N = 233^2^) | | | | | From baseline to follow-up (N = 275^3^) | | | | |
| --- | --- | --- | --- | --- | --- | --- | --- | --- | --- | --- | --- | --- | --- | --- | --- |
|  | Group * time * age | | | | | Group * time * age | | | | | Group * time * age | | | | |
| Outcome | β | 95% CI | | p_raw_ | p_cor_ | β | 95% CI | | p_raw_ | p_cor_ | β | 95% CI | | p_raw_ | p_cor_ |
| Positive affect | 0.04 | -0.01 | 0.10 | .129 | .194 | -0.10 | -0.17 | -0.03 | .004 | .024 | -0.02 | -0.09 | 0.05 | .527 | .632 |
| Internal control beliefs | 0.14 | 0.01 | 0.27 | .040 | .194 | 0.09 | -0.09 | 0.27 | .335 | .503 | 0.20 | 0.06 | 0.35 | .007 | .021 |
| External control beliefs | -0.08 | -0.17 | 0.01 | .092 | .194 | 0.04 | -0.07 | 0.16 | .470 | .564 | -0.07 | -0.18 | 0.04 | .212 | .318 |
| Self-efficacy | 0.02 | -0.10 | 0.14 | .756 | .756 | -0.01 | -0.15 | 0.13 | .930 | .930 | -0.03 | -0.16 | 0.10 | .658 | .658 |
| Favorable coping | -0.09 | -0.30 | 0.12 | .413 | .496 | -0.24 | -0.55 | 0.08 | .143 | .429 | -0.45 | -0.77 | -0.13 | .006 | .021 |
| Unfavorable coping | 0.19 | -0.04 | 0.42 | .114 | .194 | 0.20 | -0.17 | 0.57 | .285 | .503 | 0.26 | -0.06 | 0.58 | .107 | .214 |

*Note.* β = standardized beta-coefficient from multilevel mixed-effects linear regressions, adjusted for sex. CI = confidence interval. p_raw_ = uncorrected p-value. p_cor_ = corrected p-value using the Benjamini-Hochberg procedure. All outcomes were log-transformed and standardized across all waves based on the pooled standard deviation of the intervention and control group at baseline. The age variable was divided by 10 to ensure that the effects did not become too small to be presented rounded. ^1^ Participants with EMA data at baseline and/or post. ^2^ Participants with EMA data at post and/or follow-up. ^3^ Participants with EMA data at baseline and/or follow-up. The exact number of participants and observations per outcome and model is shown in Table S1.
