# Supplementary material for: Ecological momentary assessment and applied relaxation: Results of a randomized indicated preventive trial in individuals at increased risk for mental disorders
Source: PLoS One. 2023 Jun 8;18(6):e0286750. doi: 10.1371/journal.pone.0286750 (PMC10249886; doi:10.1371/journal.pone.0286750)
Supplement: S7 Table — (DOCX) [file pone.0286750.s008.docx]

Table S7

*Differences with respect to changes in other psychological outcomes from baseline to post, from post to follow-up, and from baseline to follow-up by baseline levels of the respective outcome in the intervention vs. control group (interactive effects: group * time * baseline symptom severity)*

|  | From baseline to post (N = 277^1^) | | | | | From post to follow-up (N = 233^2^) | | | | | From baseline to follow-up (N = 275^3^) | | | | |
| --- | --- | --- | --- | --- | --- | --- | --- | --- | --- | --- | --- | --- | --- | --- | --- |
|  | Group * time * baseline levels | | | | | Group * time * baseline levels | | | | | Group * time * baseline levels | | | | |
| Outcome | β | 95% CI | | p_raw_ | p_cor_ | β | 95% CI | | p_raw_ | p_cor_ | β | 95% CI | | p_raw_ | p_cor_ |
| Positive affect | -0.10 | -0.20 | 0.01 | .065 | .195 | -0.03 | -0.17 | 0.10 | .628 | .754 | -0.03 | -0.16 | 0.09 | .593 | .712 |
| Internal control beliefs | -0.24 | -0.42 | -0.07 | .006 | .036 | -0.16 | -0.40 | 0.07 | .172 | .258 | -0.42 | -0.58 | -0.26 | <.001 | <.001 |
| External control beliefs | -0.03 | -0.14 | 0.07 | .547 | .547 | 0.12 | -0.02 | 0.27 | .103 | .224 | -0.02 | -0.15 | 0.11 | .730 | .730 |
| Self-efficacy | 0.13 | -0.04 | 0.30 | .144 | .216 | -0.36 | -0.55 | -0.17 | <.001 | <.001 | -0.17 | -0.32 | -0.01 | .033 | .099 |
| Favorable coping | 0.07 | -0.13 | 0.27 | .515 | .547 | -0.03 | -0.37 | 0.31 | .871 | .871 | -0.18 | -0.45 | 0.09 | .182 | .364 |
| Unfavorable coping | 0.22 | -0.06 | 0.49 | .125 | .216 | -0.39 | -0.87 | 0.09 | .112 | .224 | -0.17 | -0.50 | 0.17 | .325 | .488 |

*Note.* β = standardized beta-coefficient from multilevel mixed-effects linear regressions, adjusted for sex and age. CI = confidence interval. p_raw_ = uncorrected p-value. p_cor_ = corrected p-value using the Benjamini-Hochberg procedure. All outcomes were log-transformed and standardized across all waves based on the pooled standard deviation of the intervention and control group at baseline. ^1^ Participants with EMA data at baseline and/or post. ^2^ Participants with EMA data at post and/or follow-up. ^3^ Participants with EMA data at baseline and/or follow-up. The exact number of participants and observations per outcome and model is shown in Table S1.
